# Neurophysiological avenues to better conceptualizing adaptive cognition

**DOI:** 10.1038/s42003-024-06331-1

**Published:** 2024-05-24

**Authors:** Jeroen Van Schependom, Kris Baetens, Guy Nagels, Simona Olmi, Christian Beste

**Affiliations:** 1 AIMS Lab, Center for Neurosciences, Vrije Universiteit Brussel, Brussels, Belgium; 2 Department of Electronics and Informatics (ETRO), Vrije Universiteit Brussel, Brussels, Belgium; 3 Brain, Body and Cognition, Vrije Universiteit Brussel, Brussels, Belgium; 4 UZ Brussel, Department of Neurology, Brussels, Belgium; 5 St Edmund Hall, University of Oxford, Oxford, United Kingdom; 6 CNR-Consiglio Nazionale delle Ricerche - Istituto dei Sistemi Complessi, Sesto Fiorentino, Italy; 7 Cognitive Neurophysiology, Department of Child and Adolescent Psychiatry, Faculty of Medicine, TU Dresden, Dresden, Germany

**Keywords:** Cognitive control, Biophysical models, Neurophysiology

## Abstract

We delve into the human brain’s remarkable capacity for adaptability and sustained cognitive functioning, phenomena traditionally encompassed as executive functions or cognitive control. The neural underpinnings that enable the seamless navigation between transient thoughts without detracting from overarching goals form the core of our article. We discuss the concept of “metacontrol,” which builds upon conventional cognitive control theories by proposing a dynamic balancing of processes depending on situational demands. We critically discuss the role of oscillatory processes in electrophysiological activity at different scales and the importance of desynchronization and partial phase synchronization in supporting adaptive behavior including neural noise accounts, transient dynamics, phase-based measures (coordination dynamics) and neural mass modelling. The cognitive processes focused and neurophysiological avenues outlined are integral to understanding diverse psychiatric disorders thereby contributing to a more nuanced comprehension of cognitive control and its neural bases in both health and disease.

## Abstract (web summary)

This article explores novel routes to better analyze neural processes underlying the brain’s adaptability and cognitive functioning, focusing on executive functions and cognitive control. It delves into the concept of “metacontrol”, proposing a dynamic balance of processes based on situational demands, and discusses the role of different concepts of neural dynamics in supporting adaptive behavior and understanding psychiatric disorders.

## Introduction

The human brain is unprecedented in its ability to flexibly adapt to varying situations and ‘behave in an intelligent way,’ which involves being able to adapt to varying demands flexibly and persist in cognitive operations when necessary. Classically, the above-mentioned aspects have, in cognitive science, been subsumed under “executive functions” or cognitive control functions^1^. These functions refer to a set of processes required for goal-directed behavior. These processes are critical for mental and physical health and are dysfunctional in many diseases and disorders.

A fundamental enigma of goal-directed behavior is what neural mechanisms make it possible to shift between thoughts from moment to moment without losing long-range goals and allow us to concentrate on a thought without getting stuck. Recent years have witnessed a flurry of studies calling from conceptual modifications of how cognitive control processes are accomplished. It has been suggested that arbitration between opposing cognitive processes, which are each important for goal-directed behavior, is accomplished through the so-called “metacontrol”^2,3^. Metacontrol can be understood as processes that are superimposed on the cognitive control functions already conceptualized in research on executive functions. Metacontrol describes the style people prefer or engage in when facing a particular situation^4^, which not only calls for cognitive functions to operate but also to operate in particular ways. Sometimes, it is useful to engage in a persistent, focused control style—like when facing distracting but irrelevant information (e.g. while working in your office), while others call for a more flexible control style—like when acting under uncertainty and being still able to monitor what is around you. Whereas a high degree of persistence corresponds to the original idea of cognitive control as willpower, with a strong focus on one goal and the information related to it, a high degree of flexibility is characterized by a more integrative, less selective, and less exclusive processing style, which facilitates switching between tasks, ideas, and actions, and taking into consideration a broader range of possibilities^2,3,5^. For example, while working in your office it is important to not dismiss the smell of smoke to stop working and better escape from the burning building. The fact that people can deal with both kinds of situations suggests that they can swiftly adjust the degree to which they engage in a specific cognitive control style^2,3,5,6^. It is especially the ease with *which* our brains can accomplish this, which is astonishing and still not understood.

It has generally been considered that synchronization and locking of oscillatory processes in electrophysiological activity are central mechanisms involved in the neural bases of cognition^7–9^. Yet, locking and synchronization can lead to “maladaptive brains because dynamics becomes locked in a state and shielded from changes in the environment”^10^. This means that the adaptive nature of goal-directed behavior cannot be fully understood by locking and synchronization processes alone. On the other hand, desynchronization (un-locking) processes allow some flexibility, but this can be a disadvantage if they prevail because long-range goals become fleeting, causing thinking to become disorganized and erratic. We discuss a potential solution to this conundrum—stressing the necessity to better connect neuroscience and psychological framings^11^—through the role of modulations in partial phase synchronization as a robust mechanism for seamlessly transitioning between thoughts while maintaining a connection with long-range goals. In physiological systems, altering the energy of a signal is time-consuming (i.e., its amplitude, power, etc.). Therefore, magnitude or energy-based mechanisms can (i) hardly explain why humans can quickly switch between opposing goals voluntarily, and (ii) can also hardly explain how such transitions can occur involuntarily. Yet, modulations in the phase properties of neural activity are less demanding, and shifting the phase of a neural signal does not inherently require energy in the sense of consuming physical resources. In this regard we delve into the possible role of neural noise for metacontrol. Moreover, using methods from “coordination dynamics”, it is possible to analyze and mathematically describe this dynamic in time series data and hence in neurophysiological data associated with processes during the unfolding of goal-directed behavior. Our contribution’s central focus lies in exploring cognitive processing along a dimension characterized by its two poles: “persistence” and “flexibility.” The continuum is also integral to understanding diverse psychiatric disorders, which is why a better conceptualization of metacontrol on a neural level will likely lead to a better mechanistic understanding of neuropsychiatric disorders. We propose signal-processing methods to characterize this continuum connecting neurophysiology, physics, and cognitive science. In this regard, also neural mass modelling is considered. While the discussion below focuses on EEG and MEG data, the different approaches discussed are of course also applicable to other form of neural time series data (e.g. invasive recordings at the level of individual neurons etc.).

## Multiple avenues for conceptualizing metacontrol on a neural level

### Neural noise

On the mechanistic level, persistence/flexibility has been related to gating or signal-to-noise ratio^12–16^. Persistence then involves amplification or maintenance of specific content in the foreground against a background of neural ‘noise,’ at the cost of less flexible responses to potentially important other signals. Interestingly, it has been suggested that this mechanism can be directly assessed through aperiodic broadband EEG activity^4^. A metacontrol bias towards persistence is achieved by increasing the top-down input of current action goals on the decision between suitable action alternatives, and by increasing the mutual inhibition (or competition) between representations of these alternatives^3^. Flexibility, in turn, would be achieved by relaxing this top-down impact and the competition between alternative representations. From a neurophysiological perspective, this suggests that some form of inhibition-excitation (I/E) balance is relevant for metacontrol and that measures reflecting this balance may be central to gaining insights into the neural underpinnings of metacontrol. Intriguingly, the I/E balance gives rise to the 1/f-like nature of the power spectral density^17^, which suggests that the 1/f-like nature of neural activity may be central to better understanding metacontrol^4^. In a nutshell, persistency-heavy processing is associated with a steeper slope of the 1/f function, while a more flexible processing strategy is associated with a flatter 1/f function. Across the EEG spectrum, a negative relationship exists between power and frequency, which can be characterized by 1*/*f^x^, with an increasing exponent (x) reflecting a steeper slope. Increasing evidence is accumulating that contextual requirements, as well as individual differences modulate 1/f slope in a manner that is consistent with an interpretation in terms of persistence/flexibility. For example, higher experimentally-induced uncertainty regarding which stimulus features are relevant for subsequent decisions induces flatter slopes, consistent with the notion that a more flexible consideration of multiple alternatives must be entertained^18^. According to this, a persistence-prone metacontrol state may be characterized by a steeper slope of the 1/f noise function and the a more flexibility-prone metacontrol state may be reflected by flatter slope of the 1/f noise function.

Critically, while evidence is rapidly accumulating that relates 1/f slope to persistence/flexibility providing a potential mechanism to metacontrol and how adaptive processes are implemented, processes captured by this measure may not be the only ones being important. The 1/f noise or other modern parameterizations, such as “fitting oscillations and 1/f (FOOOF)”^17^, are applied to measure broadband noise. Higher frequency band activity contributes to 1/f noise (e.g., from the gamma frequency band). While there is some evidence that 1/f-like dynamics is evident in narrow-band amplitude fluctuations^19,20^, this dynamics is likely mostly a side-effect of broadband activity. The reason is that low-frequency oscillations are co-modulated with higher frequencies by phase-amplitude coupling^8,21–23^. From that perspective, it is unlikely that a 1/f regimen alone is sufficient to ‘fully’ conceptualize metacontrol on a neurophysiological level. It is thus necessary to consider other potential mechanisms related to oscillatory dynamics and their biophysics. Importantly, this possible dynamic has to forego the problem by synchronization (locking) and desynchronization (un-locking).

### Transient brain dynamics

Aside from “noise” or aperiodic activity, recent modelling and conceptual advances have led to the characterization of oscillatory activity as a series of bursts in specific networks rather than continuous ongoing activity.

One interesting aspect is so-called “phase precession”. It is a phenomenon observed in the firing patterns of neurons and refers to a pattern where the spiking firing pattern of neurons shift their timing relative to ongoing oscillations (e.g. the theta frequency band). Neural ensembles revealing phase precession properties are able to fire in a phase-locked (persistent) state, when there are no changes in the associated cognitive state. Yet, these ensembles also exhibit changes (flexibility) in their phases of oscillations as the cognitive state is updated through environmental interactions. While originally described in the hippocampus^24,25^, phase precession has also been found in the medial prefrontal cortex^26^ and other cortical regions^27,28^. This implies a more global role for this phenomenon during theta rhythm-mediated coordination of neural activity ^27^. Interestingly, especially the medial prefrontal cortex is well-known to be involved in cognitive control and relevant for adaptive cognitive processes^29,30^ and the same holds true for theta band activity^7,31^. Therefore, phase precession could reflect an important mechanism through which adaptive cognitive processes may be realized. In particular it is conceivable that a flexibility-prone state is reflected by a pattern where spiking neural activity is not closely tied to the timing of ongoing oscillations. In a persistence-prone state, the opposite may be the case.

**Fig. 1:**
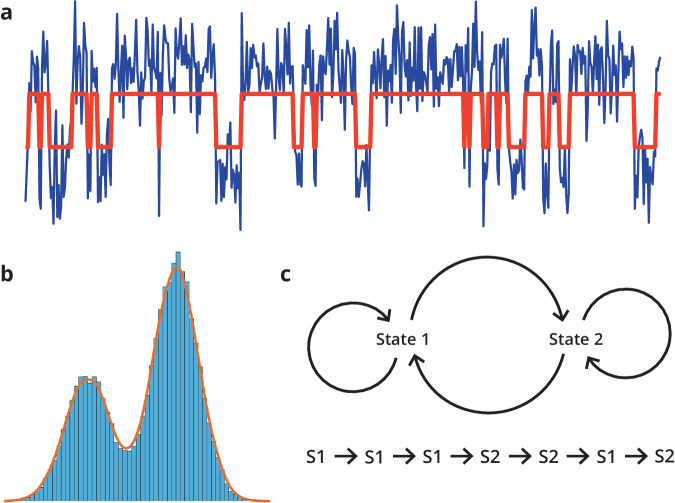
Example of an HMM application. A single time series (panel **a**, blue line) consists of a multimodal distribution as visualized by the histogram in panel **b**. The underlying Markov chain is visualized in panel **c**. The system cycles through two states, and each state has an observation model, which - in this case - is a univariate random Gaussian distribution characterized by its mean and standard deviation. Inferring the HMM refers to uncovering the states’ parameters and their activations (red line in panel **a**).

**Fig. 2:**
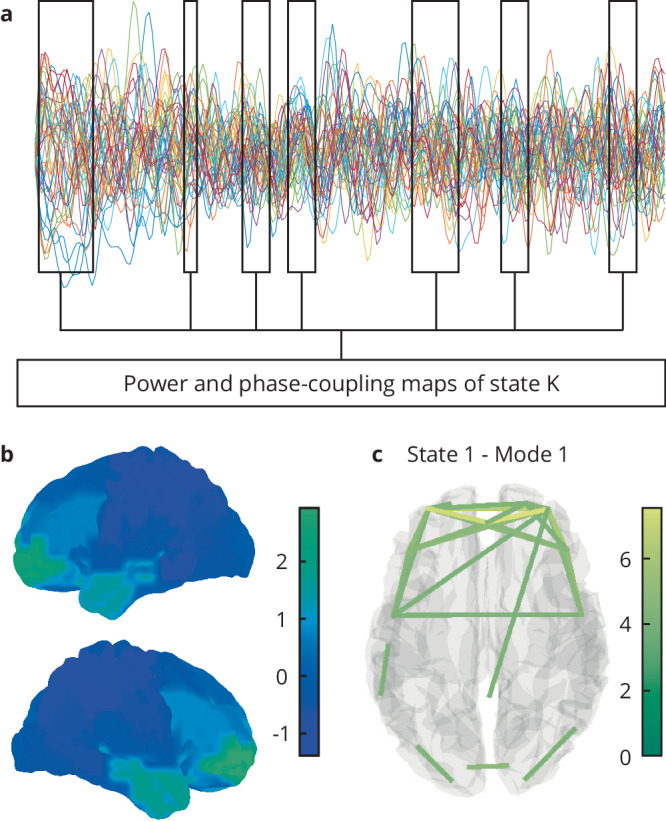
Example of the application of HMM models to source-reconstructed MEG data. The HMM model identifies the time points at which a certain state K is active (panel **a**). This HMM state can be further characterized by its power spectral map (**b**) and its functional connectivity pattern (**c**).

Another line of research, also applicable using surface-based neurophysiological data (e.g. EEG/MEG), is using novel modelling approaches to capture and characterize fast brain dynamics. One such approach is based on hidden Markov models (HMMs). These models assume that the brain’s activity progresses through different states that are hidden and Markovian. The observation model then associates e.g. a mean brain activity across different brain regions to each of the states. Put more concretely, this modelling approach starts from fMRI or source-reconstructed M/EEG data and extracts several brain ‘states’. These states have a spatial profile, can be characterized by specific frequency content, and (in the case of M/EEG) allow us to analyze the dynamics at millisecond resolution, i.e., these brain states have lifetimes in the order of 50–100 ms. These methods have been used to characterize the effect of normal brain functioning, benzodiazepines^32^, or working memory in health^33^ and disease^34^. Given that these models capture fast transient brain dynamics and can capture the stability of specific brain networks, they are excellent candidates for distinguishing people on the persistence vs flexibility axis. Hidden Markov models (HMMs) have been used in a variety of settings to uncover hidden sequences underlying observations. One example to illustrate the use of hidden Markov models goes as follows (see Figs. 1 and  2): assume you have a single variable that fluctuates over time with a certain mean and standard deviation. However, when plotting the histogram of values, you observe that the distribution does not resemble a simple unimodal distribution but seems to present different modes. Inferring the hidden Markov model on this data would then boil down to discovering the underlying states, their parameters (in this case, the mean and standard deviation of the normal distribution), and transition probabilities. Put concretely, if you have one EEG time series captured during sleep, you could use the HMM framework to extract the different sleep stages. An excellent starting point to understand hidden Markov models can be found elsewhere^35^. To model whole-brain activity, it is necessary to have a (predefined) number of states and an observation model that links these states to the observed brain activity. There are several options for the observation model. One of the first HMM models used a multivariate normal distribution, where each state was characterized by its mean activation and the covariance between the different time series^36^. More advanced models have been developed: the multivariate autoregressive model enables to model of time-lagged dependencies^37^ but is computationally unfeasible on whole-brain imaging data. Therefore, Vidaurre et al. developed the time-delay embedded HMM model. Here, the original time courses are supplemented with time-delayed versions. Next, a principal component analysis is applied to extract the main principal components, and a multivariate normal distribution is used as observation model^38^. The hidden brain states allow us to characterize brain activity in whole-brain networks that are transiently activated and that have an associated frequency spectrum. As such, one can compare the 1/f-spectrum per brain state between two conditions or the transition probabilities between different hidden states. Concerning metacognition, one could hypothesize that flexibility vs persistence could be represented by the stability of the different brain states as captured by Markovian processes. Regarding metacontrol, an HMM provides parameters of the underlying states (e.g. of the stability of the different brain states), and its transition probabilities. For a flexibility-prone state the parameters indicate a lower stability of the different brain states and higher transition probabilities. For a persistence-prone state the HMM is likely to yield a stronger a stability of the different states and lower transition probabilities.

However, aside HMM models to capture transient brain dynamics, also other concepts have been around that may provide suitable means to capture transient dynamics. One of these concepts is “chaotic itinerancy”^39,40^. In a nutshell, this concept suggests that transitions between cortical activity states are not random but yield chaotic dynamics^39,40^. As such, chaotic itinerancy generates metastable states^40,41^. As outlined in the introduction, these are central because locking and synchronization can lead to “maladaptive brains because dynamics becomes locked in a state and shielded from changes in the environment”^10^. Through “chaotic itinerancy”^39,40^, such problems can be avoided. Central to this concept is the existence of so-called quasi-attractors. The latter reflects a region giving ordered (periodic) and disordered, chaotic activity^39^. Of note, it has been argued that such states can be observed in networks showing scale-free properties of activity^40^. This is important because scale-free activity is fundamental to the emergence of 1/f noise or aperiodic activity (see above)^42^. The concept of chaotic itinerancy could thus provide links between transient dynamics and noise approaches to better conceptualized adaptive cognitive and metacontrol. Regarding metacontrol and the arbitration between a flexibility-prone and a persistence-prone state their effect on chaotic itinerancy may be as follows: Chaotic itinerancy assumes a closed-loop trajectory through high-dimensional state space of neural activity that directs the cortex in a sequence of quasi-attractors^39^. Within a flexibility-prone state this quasi-attractor is more governed by disordered chaotic activity between regions, while in a persistence-prone state this quasi-attractor is governed by ordered periodic activity. This, however, means that there is a dynamic interplay or periodic and chaotic (aperiodic) activity to establish the dynamic interplay of cognitive state as supposed by metacontrol theory. Both, periodic and aperiodic activity, therefore need to be considered in parallel to capture metacontrol dynamics.

### Phase-based measures

**Fig. 3:**
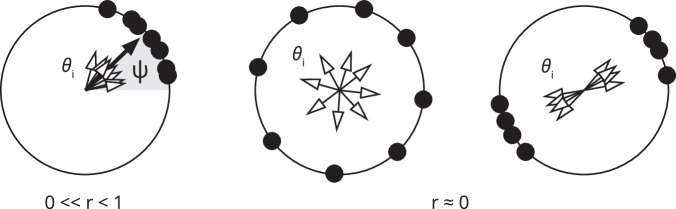
Illustration of Kuramoto order parameter background. The Kuramoto order parameter (1.3) encodes the level of synchrony of a phase oscillator population. The state of each oscillator is given by a phase θi (black dot, empty arrow) on the circle T. The left part of the figure shows a configuration with high synchrony where r = |Z| ≈ 1. The middle part of the figure shows a configuration with r = |Z| ≈ 0 where the oscillators are approximately uniformly distributed on the circle. Also the right part of the figure shows a configuration of oscillations with r = |Z| ≈ 0, which is organized into two clusters.

As outlined above, a characterization of the phase properties of neural activity can be key to the conceptualization of adaptive cognitive processes. The framework of phase-based signal analysis offers a means to characterize the dynamics underlying experimental neural signals. A decomposition of signals into instantaneous amplitudes and instantaneous phases can readily be achieved using the so-called analytical signal approach based on the Hilbert transform. The instantaneous phases can be used to characterize individual signals, pairs of signals, or multivariate sets of signals, thereby covering different spatial scales of neuronal organization. For individual signals, the degree of regularity versus irregularity can be quantified by the coefficient of phase velocity variation^43^. This approach allows one to assess the synchronization of local ensembles of neurons contributing to the signals measured at individual M/EEG sensors. The synchronization between pairs of neuron ensembles measured at pairs of M/EEG sensors can be quantified by the mean phase coherence^44^. The mean resultant length^45^, as well as its renormalized definition^46^, can be used to quantify the overall phase coherence of an extended network as measured by a multitude of M/EEG sensors. In particular, these latter multivariate approaches can assess a network’s instantaneous phase coherence, thereby offering a glance at the two poles of persistence and flexibility in cognitive processing. Next to the analysis of whole-brain network bursts, different measures can capture the intrinsic stability of neurophysiological signals. One of them is the Kuramoto Order parameter (see Box 1). This parameter quantifies the degree of phase-locking in an ensemble of oscillators at any time; importantly, it captures the degree of phase coherence in the system as it vanishes when the phases are uniformly distributed and approaches one when the phases of all oscillators become aligned, thus covering the overall structure. An extension of the classical Kuramoto Order parameter to account for higher harmonics of phases allows considering another type of phase locking, i.e. the formation of symmetric phase clusters, where the oscillators in a network separate into groups, each one oscillating in different phases. When a network is stable, we expect the Kuramoto order parameter to remain (more or less) constant. However, if other networks start competing, the Kuramoto order parameter may start to increase or decrease. Understanding how the Kuramoto order parameter evolves within trials, across trials, and subjects and how it is related to bursting network activity (see above) provides a first promising approach toward a novel characterization of Metacontrol in healthy and pathological brain functioning. In addition, characterizing the Kuramoto order parameter not across the whole brain but across the well-known subnetworks of the brain would further help elucidate the stability and flexibility of each subnetwork. Through the Kuramoto order parameter, one could derive a measure for a candidate neural mechanism possibly consistent with this well-known, yet unexplained nature of goal-directed behavior, which is an “intermediate” of the locking (synchronization) and un-locking (desynchronization) processes and be considerably dynamic to become fully effective in short time frames. Instead of research on goal-directed behavior conventionally centering on mechanisms of neural locking (synchronization) or unlocking (desynchronization), the use of the Kuramoto Model may be useful to better understand the dynamics of goal-directed behavior. Interestingly, the Kuramoto model has already been regarded to reflect “metastability”, which is the simultaneous realization of two competing tendencies: the tendency of the individual components (oscillators in a network) to couple together and the tendency for the components to express independent behavior. The concurrent expression of both temporary large-scale integrative activity and local autonomous activity is achieved via partial coordination or patterns of quasi-phase-locking that summon and release brain areas “on demand”. Using methods from “coordination dynamics” (i.e., the Kuramoto model), it may be possible to analyze and mathematically describe metastability in time series data and hence in neurophysiological data associated with processes during the unfolding of goal-directed behavior. The Kuramoto order parameter encodes the level of synchrony of a phase oscillator population and could be used to quantify the degree as to which a system is in a persistence-prone or flexibility-prone state. A Kuramoto order parameter close to one indicates a high level of synchrony of a phase oscillator population, which could give rise to a persistence-prone metacontrol state. Vice versa, a Kuramoto order parameter closer to zero could indicate a more flexibility-prone metacontrol state. Higher Kuramoto order parameters (see also Box 1 and Fig. 3) may finally disclaim the case where there is only one flexibility-prone metacontrol state or several persistence-prone metacontrol states.

Box 1 Phase oscillators and the Kuramoto Order Parameter A phase oscillator network can be imagined as an ensemble of nodes, where the state of each node is given by a single-phase variable. More specifically, consider a population of N oscillators where the state of an oscillator (i) is given by a phase θ_i_ ∈ T, where T indicates the circle of all phases [0, 2π). Without input, the phase of each oscillator (i) advances at its intrinsic frequency ω_i_ ∈ R. The input to the oscillator (i) is determined by a complex field H_i_ (t), which is modulated by a sinusoidal function; this field could be due to an external driving or to network interactions between oscillators both within the same population or other populations. In other words, we consider oscillator networks whose phases evolve according to

1.1 $${{{{{{\rm{d}}}}}}{{{{{\rm{\theta }}}}}}}_{{{{{{\rm{i}}}}}}}/{{{{{\rm{dt}}}}}}={{{{{{\rm{\omega }}}}}}}_{{{{{{\rm{i}}}}}}}+{{{{{\rm{Im}}}}}}({{{{{{\rm{H}}}}}}}_{{{{{{\rm{i}}}}}}} \, ({{{{{\rm{t}}}}}}) \, {{{{{{\rm{e}}}}}}}^{-{{{{{\rm{i}}}}}}{{{{{\rm{\theta }}}}}}{{{{{\rm{i}}}}}}} \, )$$

While we allow the intrinsic frequency and the driving field to depend on the oscillator to a certain extent (i.e., they are nonidentical), we will henceforth also assume that all oscillators within any given population are indistinguishable: this means that the properties of each oscillator in a given population are determined by the same distribution. Specifically, suppose that the properties of each oscillator are determined by a parameter η_i_ - for example, the excitability in the case of a QIF neuron described in the next text box. Now let both the intrinsic frequencies and the field be functions of this parameter, that is, ω_i_ = ω(η_i_) and H_i_ (t) = H(t; η_i_). The oscillators of a given population are *indistinguishable* if all η_i_ are random variables sampled from a probability distribution with density g(η). In the special case that all η_i_ are equal (i.e. g is a delta-distribution) the oscillators are *identical*. If η_i_ is not equal, the oscillators are not identical or *heterogeneous*.

Phase oscillator networks of the form (1.1) include a range of well-known models, such as the Kuramoto model, which consists of a population of N coupled phase oscillators, θ_i_ (t), having natural frequencies ω_i_ distributed with a given probability density g(ω), and whose dynamics is governed by:

1.2 $${{{{{{\rm{d}}}}}}{{{{{\rm{\theta }}}}}}}_{{{{{{\rm{i}}}}}}}/{{{{{\rm{dt}}}}}}={{{{{{\rm{\omega }}}}}}}_{{{{{{\rm{i}}}}}}}+{\sum }_{{{{{{\rm{j}}}}}}=1}^{{{{{{\rm{N}}}}}}}{{{{{{\rm{K}}}}}}}_{{{{{{\rm{ij}}}}}}}\sin ({{{{{{\rm{\theta }}}}}}}_{{{{{{\rm{j}}}}}}}{{{{{\rm{\hbox{-}}}}}}}{{{{{{\rm{\theta }}}}}}}_{{{{{{\rm{i}}}}}}}) \, {{{{{\rm{i}}}}}}=1,\ldots ,{{{{{\rm{N}}}}}}$$

Thus, each oscillator tries to run independently at its frequency, while the coupling tends to synchronize it with all the others. When the coupling is sufficiently weak, the oscillators run incoherently, whereas beyond a certain threshold, collective synchronization emerges spontaneously. Many different models for the coupling matrix K_ij_ have been considered such as nearest-neighbor coupling, hierarchical coupling, random long-range coupling, or even state-dependent interactions. If all entries of the coupling matrix are identical and equal to 1, all oscillators are identically coupled to all the others, and the network is said to be *globally coupled*. The synchronization transition observable in the model (1.2) can be evaluated once the (complex-value) order parameter (known as the Kuramoto Order Parameter)

1.3 $${{{{{\rm{Z}}}}}}={{{{{\rm{r}}}}}} \, {{{{{\rm{e}}}}}}^{{{{{{\rm{i}}}}}}{{{{{\rm{\psi }}}}}}}=1/{{{{{\rm{N}}}}}}{\sum }_{{{{{{\rm{j}}}}}}=1}^{{{{{{\rm{N}}}}}}}{{{{{{\rm{e}}}}}}}^{{{{{{\rm{i}}}}}}{{{{{\rm{\theta }}}}}}{{{{{\rm{j}}}}}}}$$

that is the mean of all phases on the unit circle. Here r(t) with 0 ≤ r(t) ≤1 measures the coherence of the oscillator population. Its magnitude r =|Z| describes the level of synchronization of the oscillator population, see Fig. 3: on the one hand, r = 1 if and only if all oscillators are phase synchronized, that is, θ_k_ = θ_j_ for all k and j; on the other hand, we have r = 0 for example if the oscillators are evenly distributed around the circle. The argument ψ of the Kuramoto order parameter Z describes the ”average phase” of all oscillators, that is, it describes the average position of the oscillator crowd on the circle of phases.

As it turns out to be clear in Fig. 3, when looking at the middle and right panels, the Kuramoto order parameter cannot differentiate between completely asynchronous behaviors (where phases may be randomly distributed on the unit circle) and synchronized behaviors with multiple symmetric phase clusters (where each cluster or group consists of fully synchronized oscillators, and different groups are phase-locked with nonzero phase difference). In other words, the Kuramoto order parameter Z is sufficient to measure the synchrony provided that the coupling function does not include any higher-order phase harmonics. To overcome this limitation, it is convenient to introduce the Kuramoto-Daido order parameters, which represent mean-fields of the higher harmonics of phases:

1.4 $${{{{{{\rm{Z}}}}}}}_{{{{{{\rm{m}}}}}}}={{{{{{\rm{r}}}}}}}_{{{{{{\rm{m}}}}}}} \, {{{{{{\rm{e}}}}}}}^{{{{{{\rm{i}}}}}}{{{{{\rm{\psi }}}}}}{{{{{\rm{m}}}}}}}=1/{{{{{\rm{N}}}}}}{\sum }_{{{{{{\rm{j}}}}}}=1}^{{{{{{\rm{N}}}}}}}{{{{{{\rm{e}}}}}}}^{{{{{{\rm{im}}}}}}{{{{{\rm{\theta }}}}}}{{{{{\rm{j}}}}}}},{{{{{\rm{m}}}}}}=1,\ldots .,4,$$

with Z_m_ being the centroid of N points {e^imθ j^} on the unit circle in the complex plane. For *m* = 1 one obtains again Eq. (1.3) (i.e. Z_1_ ≡ Z), while for m = 2 it is possible to take into account the formation of two clusters separated by phase difference π. Referring to Fig. 3, r _2_ = 0 if the oscillators are evenly distributed around the circle, while r_2_ = 1 in the case shown in the right panel. Therefore, the Daido order parameter Z_2_ is needed to quantify the overall degree of synchronization via entrainment in both clusters, while Z_1_ just measures the degree of asymmetry between the clusters. Equivalently for m = 3 (m = 4) the order parameter Z_3_ (Z_4_) identifies the formation of 3 (4) symmetrically formed clusters.

### Neurocomputational approaches

Whereas signal processing techniques can help us characterize brain functioning across a range of neuropsychiatric disorders, it is important to remember that the signals we can measure noninvasively are necessarily averages across large neuronal populations. Neurocomputational models, however, have the potential to explain these changes or—at least—to generate new hypotheses on how Metacontrol emerges and is altered across normal and pathological functioning. In the cortex, neural activity is coordinated across the network, despite weak, zero-lag cross-correlations between pairs of neurons. As a result, the collective network dynamics can be effectively described by a small number of collective variables when compared to the number of (simultaneously) recorded neurons. These collective variables are typically obtained using dimensionality-reduction techniques such as principal component analysis. It has been proposed that low-dimensional dynamics are a consequence of the fact that the connectivity matrix is rank-deficient^47^, which seems to require either some form of synaptic plasticity or some specific anatomical organization of the connectivity. In either case, the biological underpinning of the postulated low-rank connectivity remains unidentified.

Neural mass and field models, able to generate brain rhythms using the notion of population firing rates, have been usually employed to reproduce the above-mentioned low-dimensional collective dynamics of the active cortex. While more detailed networks of interacting conductance-based spiking neuron models are hard to analyze in the raw data, given that they are both high-dimensional and nonlinear, neural mass and field models are much more amenable to mathematical analysis, as reviewed in Cook et al. ^48^. Even though these low-dimensional models are typically not derived from any underlying microscopic spiking dynamics, they can be motivated by several phenomenological arguments for the evolution of coarse-grained neuronal variables. The population model of Wilson–Cowan^49,50^ is perhaps the most well-known among these neural mass and field models that can be derived from underlying microscopic dynamics, but do not come from a biophysically detailed description of a spiking neuron: a core part of its modelling framework is the use of a sigmoid function to determine population firing rates in terms of population activity, while, at the tissue level, the spatially extended Wilson–Cowan model can be conceived of as a (spatially continuous) network of neural masses describing population activity where a form of non-local spatial interaction is adopted to describe anatomical connections and signaling along axonal fiber tracts.

Nonetheless, they are only expected to provide appropriate levels of description for many thousands of near-identical interconnected neurons with a preference to operate coherently. As a consequence, they are not ideally suited to studying phenomena that are known to be associated with changes of synchrony, such as the post-stimulus response ubiquitously seen in human neuroimaging studies^51^ and which are currently focused on debates on how adaptive behavior is accomplished. Recently, a new type of neural mass model has been developed that can capture the phenomenon of event-related synchronization/desynchronization (ERS/ERD) that is believed to underlie the changes in power seen in brain spectrograms. Importantly, this new mean-field model is an exact description of a network of non-identical (heterogeneous) θ-neuron models in the limit of infinitely many neurons, where all the neurons are connected and interacting among them in a symmetric manner (globally coupled). The θ-neuron, or Ermentrout–Kopell canonical model (see Box 2), is the normal form for the saddle-node on a limit cycle bifurcation and for, constant stimulation, can generate low firing rates typical of those seen in real cortical neurons^52^. Interestingly, the resulting mean-field model has a population firing rate that depends on the degree of population synchrony, measured by the Kuramoto order parameter^53–55^, rather than just keeping track of the fraction of active neurons in a given interval of time (as in the original heuristic derivation of the Wilson–Cowan equations). The inclusion of a dynamic component for describing spike synchrony enables tracking within-population synchrony in a tractable way. Moreover, this mean-field model can also be expressed in terms of a few collective variables representing the firing rate and the mean membrane potential of the neuronal populations^56,57^. In all cases, it can incorporate realistic models of synaptic currents (both chemical and electrical), and it can be easily embedded into network studies (both discrete and continuous). Thus, we may select from one of the two equivalent perspectives depending on context. For example, when modelling MEG studies that highlight ERS/ERD it is convenient to use the Kuramoto order parameter representation, while it is natural to use the membrane potential form when calculating the power spectrum of the local field potential.

**Fig. 4:**
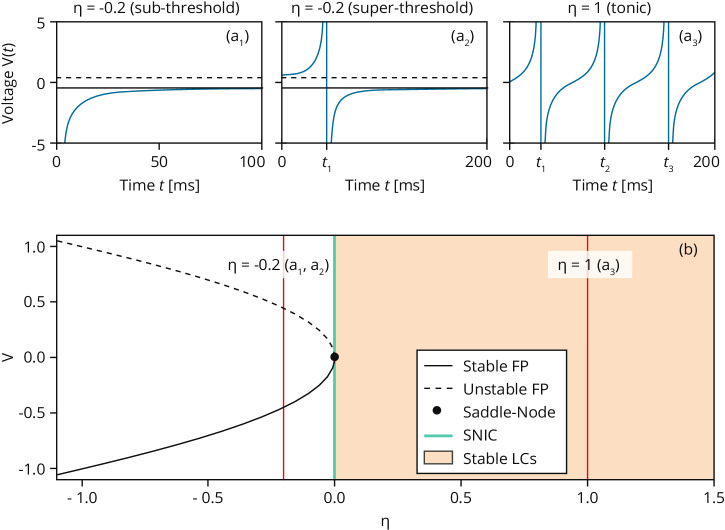
Dynamics of a single QIF neuron. (**a**1) shows the excitable case with V(0) <√|η | , (**a**2) with V(0)>√|η| and (**a**3) corresponds to tonic firing. The solid and dashed black lines mark V = ∓ √|η| and spike times are denoted by t1, t2, and t3. (**b**) Bifurcation diagram V versus η. The solid and dashed black lines show the stable and unstable fixed points of Eq. (2.2). The saddle-node bifurcation at (V = 0, η = 0) is marked by a black dot; the SNIC bifurcation at η = 0 by a green line. The orange region marks η ≥ 0, for which stable limit cycles exist.

As a result, this new type of neural mass model has a richer dynamical repertoire than standard neural mass models in terms of macroscopic behavior. For example, a heuristic firing rate model, specifically designed to reproduce the QIF network dynamics (see Fig. 4) with short-term plasticity, does not display any oscillatory activity in the *β*-*γ* range, contrary to what is observable in the spiking network itself and the equivalent neural mass model^58^.

In general, a key feature of this new type of neural mass model is the ability to capture transient synchronization properties and oscillatory dynamics present in the spiking networks, that are lost in usual rate models. In particular, with this new type of neural mass and neural field models are observable phenomena such as multi-stability, the coexistence of oscillatory and non-oscillatory behaviors, and various behaviors displaying multiple time scales. The wide variety of reachable states, together with the ability of the model to describe systems that dynamically evolve between an incoherent state and a partially synchronized state seems to be the ideal mathematical framework for understanding the role of modulations in partial phase synchronization and Metacontrol related processes.

In this direction, advances in non-invasive neuroimaging methods that allow for a detailed characterization of the brain’s anatomy and activity, together with developments in network science, have supported a proliferation of network connectivity-based approaches, employing neural mass models as building blocks, to understand large-scale brain function. The advantage of using an exact description of a network of heterogeneous θ-neurons in the infinite size limit as a neural mass model lies in the possibility of an exact (analytical) moving upwards through the scales, while keeping the influence of smaller scales on larger ones it levels out their inherent complexity. At the same time, moving downwards through the scales, more detailed modelling parameters can be used, e.g. to test specific hypotheses. In particular, having a 1:1 correspondence between microscopic (i.e. neuronal), mesoscopic (i.e. single population), and macroscopic (i.e. network) levels will help us to understand at which level it is most appropriate to test the action of the Metacontrol.

Network studies are especially relevant to elucidating the emergence of functional connectivity networks that describe dynamic patterns of temporal coherence of activity between brain regions. Important examples include archetypal brain networks that emerge under different tasks or stimulants^59^, and so-called resting state networks^60^, whereby different regions of the brain’s sensorimotor system oscillate slowly and synchronously in the absence of any explicit task. More generally, these functional connectivity networks are implemented to support high-level brain function, while the divergence between dynamic functional activity and the relatively static structural connections between populations turns out to be critical to the brain’s wide functional repertoire and may hold the key to understanding brain activity in health and disease^61–63^. However, while functional connectivity is widely employed in both empirical and theoretical studies, the specific link between the brain’s anatomical circuitry and the varied and complex behavior it exhibits is not fully understood^61,64^. In particular, the application of a (next generation) neural mass network model, incorporating human connectome data to relate structural and functional connectivity, can be used to investigate the relationship between the disruptions in structural and functional brain networks and a variety of psychiatric and neurological diseases^65–67^. While the initial formulation of the neural mass model^56,57^ has been developed for globally coupled deterministic populations, a recent formulation can encompass further fundamental aspects of brain circuits beyond heterogeneity, as sparseness in the synaptic connections (as opposite to global coupling) and background noise^68^, thus being able to reproduce spiking network dynamics induced by various noise sources. Within this formalism, we could also reproduce the 1/f power spectral structure of LFPs, as well as more complex frequency scaling to understand whether the 1/f slope is dependent on task context. Finally, the model shows the possibility of supporting collective oscillations in different frequency bands as well as coupling between neural oscillations at different timescales^69–71^. Thus, through the use of neural mass models, “neural noise” approaches, approaches reflecting “transient brain dynamics” and “phase-based” measures of dynamics may be combined and lead to a harmonized formulation of neural dynamics underlying Metacontrol. Neural mass models offer a means to formalize insights gained from testing the hypotheses outlined above on how a flexibility-prone or a persistence-prone state affects neural noise or measures of transient dynamics.

Box 2 Simplified neuronal models and mean-field formulation The Ermentrout-Kopell canonical model is better known as the “θ-model” and is a simple one-dimensional model for the spiking of a neuron: it describes a single neuron using a phase θ ∈[0, 2π) such that a spike is generated whenever θ passes through π from below. The one-dimensional ordinary differential equation

2.1 $${{{{{\rm{d}}}}}}{{{{{\rm{\theta }}}}}}/{{{{{\rm{dt}}}}}}=1-{{{{\mathrm{cos}}}}}\; {{{{{\rm{\theta }}}}}}+(1+{{{{\mathrm{cos}}}}}\; {{{{{\rm{\theta }}}}}}){{{{{\rm{I}}}}}}$$

describes the universal dynamic that occurs on the invariant circle near an SNIC bifurcation (a global bifurcation in which multiple structures interact, such as heteroclinic orbits, fixed points, etc.) in at least a two-dimensional space. The variables θ and I in (2.1) represent the dimensionless membrane potential and current injected into the neuron. In this representation, neurons are seen as simple phase oscillators characterized by an angular variable. When I < 0, the system is excitable, i.e., given an appropriate perturbation, the system will produce a spike. When I > 0, dθ/dt is also positive, and the system will give rise to a limit cycle (periodic oscillating behavior). For I = 0, the equilibrium points present for I < 0 merge exactly and disappear for I > 0.

The $\Theta$-model is often presented with another name: the Quadratic Integrate-and-Fire (QIF) model. The QIF model is usually preferred because of its natural interpretation in terms of the membrane potential of the neuron. The equation governing the evolution of the QIF model can be obtained from the (2.1), under the transformation V = tan(θ/2):

2.2 $${{{{{\rm{dV}}}}}}/{{{{{\rm{dt}}}}}}={{{{{{\rm{V}}}}}}}^{2}+{{{{{\rm{\eta}}}}}} \; {{{{{\rm{with}}}}}}\; {{{{{\rm{the}}}}}}\; {{{{{\rm{reset}}}}}}\; {{{{{\rm{rule}}}}}}:{{{{{\rm{if}}}}}}\;{{{{{\rm{V}}}}}} \, > \, {{{{{{\rm{V}}}}}}}_{{{{{{\rm{thresh}}}}}}}:{{{{{\rm{V}}}}}}\leftarrow {{{{{{\rm{V}}}}}}}_{{{{{{\rm{reset}}}}}}}$$

The parameter η (that replaces I in 2.1) represents a constant external current that determines the neuronal excitability. The QIF neuron exhibits two possible dynamics, depending on the sign of η (see Fig. 4).

To investigate the emerging dynamics of interacting neurons, one can consider an ensemble of synaptically coupled QIF neurons. In such a network of i = 1, … N QIF neurons, the dynamics of the system reads

2.3 $${{{{{\rm{d}}}}}}{{{{{{\rm{V}}}}}}}_{{{{{{\rm{i}}}}}}}/{{{{{\rm{dt}}}}}}={{{{{{\rm{V}}}}}}}_{{{{{{\rm{i}}}}}}}^{2}+{{{{{{\rm{\eta }}}}}}}_{{{{{{\rm{i}}}}}}}+1/{{{{{\rm{N}}}}}}{\sum }_{{{{{{\rm{j}}}}}}=1}^{{{{{{\rm{N}}}}}}}{{{{{{\rm{J}}}}}}}_{{{{{{\rm{ij}}}}}}}\left({{{{{\rm{t}}}}}}\right){{{{{{\rm{S}}}}}}}_{{{{{{\rm{j}}}}}}}({{{{{\rm{t}}}}}})+{{{{{{\rm{I}}}}}}}_{{{{{{\rm{s}}}}}}}({{{{{\rm{t}}}}}}),$$

where I_s_(t) is an external time-dependent current, while J_ij_ (t) represents the strength of the direct synapse from neuron j to i that, in the absence of plasticity, we assume to be constant in time and all identical, i.e. J_ij_ (t) = J. The sign of J determines if the neurons are excitatory (J > 0) or inhibitory (J < 0). Moreover, the excitability parameters η_i_ can be heterogeneously distributed according to a certain probability distribution. Now the total synaptic current due to the recurrent connections with presynaptic neurons reads s(t) = 1/N ∑^N^_j=1_ S_j_(t) and we can rewrite the dynamics as:

2.4 $${{{{{\rm{d}}}}}}{{{{{{\rm{V}}}}}}}_{{{{{{\rm{i}}}}}}}/{{{{{\rm{dt}}}}}}={{{{{{\rm{V}}}}}}}_{{{{{{\rm{i}}}}}}}^{2} \, ({{{{{\rm{t}}}}}})+{{{{{{\rm{\eta }}}}}}}_{{{{{{\rm{i}}}}}}}+{{{{{{\rm{J}}}}}}}_{{{{{{\rm{s}}}}}}}({{{{{\rm{t}}}}}})+{{{{{{\rm{I}}}}}}}_{{{{{{\rm{s}}}}}}}({{{{{\rm{t}}}}}}).$$

In the following, we restrict to the case of instantaneous synapses, for which the total synaptic current s(t) is identical to the instantaneous mean firing rate of the network, i.e. s(t) = r(t). In the infinite size limit, it is possible to reproduce the collective evolution of a single population of QIF neurons (2.4) with two collective variables representing the firing rate and the mean membrane potential of the neuronal populations:

2.5 $${{{{{\rm{dr}}}}}}/{{{{{\rm{dt}}}}}}=\varDelta /{{{{{\rm{\pi }}}}}}+2{{{{{\rm{\pi }}}}}}{{{{{\rm{rv}}}}}},{{{{{\rm{dv}}}}}}/{{{{{\rm{dt}}}}}}={{{{{{\rm{v}}}}}}}^{2}+{{{{{\rm{\eta }}}}}}+{{{{{\rm{Jr}}}}}}+{{{{{{\rm{I}}}}}}}_{{{{{{\rm{s}}}}}}}({{{{{\rm{t}}}}}})-{{{{{{\rm{\pi }}}}}}}^{2}{{{{{{\rm{r}}}}}}}^{2}$$

where r is the population firing rate, v is the average membrane potential, J is the synaptic weight, I_s_ an external current applied to all neurons and η represents the average neural excitability (for η < 0 (η > 0) the neuron is sub- (supra-) threshold). The term Δ is the width of the distribution of the neural excitabilities, therefore this parameter controls the heterogeneity of the population. The exact derivation of Eq. (2.5) from Eq. (2.4) is shown in Montbrió et al. ^57^. Moreover, it is possible to derive an exact neural field model starting from a single population of spiking Θ-neurons, thus reproducing the collective evolution of the population with two collective variables representing the level of synchronization of the population, in terms of the amplitude of the Kuramoto order parameter and average phase of all the oscillators^53–55^.

### Metacontrol and Psychopathology

The conceptualization of persistence versus flexibility as a dispositional metacontrol bias has substantial explanatory power in explaining information processing characteristics associated with diverse forms of psychopathology. Prime examples are obsessive-compulsive disorder (OCD) and attention deficit hyperactivity disorder (ADHD), representing extremes on the persistence and flexibility end of the spectrum, respectively^14^. Underscoring the explanatory power of this account, it cannot only organize the findings regarding cognitive deficits associated with such descriptive diagnoses (e.g., perseverative errors in a rule inference task such as the Wisconsin Card Sorting Test – OCD, versus problems with sustained attention - ADHD) but can also do right by findings regarding related cognitive strengths (e.g., superior divergent thinking in ADHD)^14^. Beyond these hallmark examples, bias towards the flexibility end can, for example, be recognized in positive thought disorder in schizophrenia, i.e., broader spreading of activation in semantic networks^72^, or in the association between schizotypy and divergent thinking^73^. In anxiety, depression, and post-traumatic stress, repetitive negative thoughts (worries, traumatic intrusions, rumination) may reflect an undermined ability to maintain a focus on other mental content^74–76^, in keeping with reported deficits in inhibitory control in these populations^77–79^. The restricted, repetitive patterns of behavior observed in autism could be an example of a persistence bias, which dovetails with congruent cognitive strengths in this population, such as superior visual search among distractors^80^. However, it is critical to take a sufficiently dynamic perspective in this respect, as apparent in the case of gambling and substance addiction. While there is a larger risk of developing such problems in individuals with a flexibility bias, e.g., ADHD^81–83^, the addiction process itself gives rise to persistent, compulsive behavior and response perseveration^84^. Indeed, metacontrol as a relevant interindividual characteristic is not to be simplistically understood as an all-pervasive bias towards one end of the persistence/flexibility dimension, but also as the tendency or ability to shift between these poles in a more or less adaptive manner concerning situational demands.

**Fig. 5:**
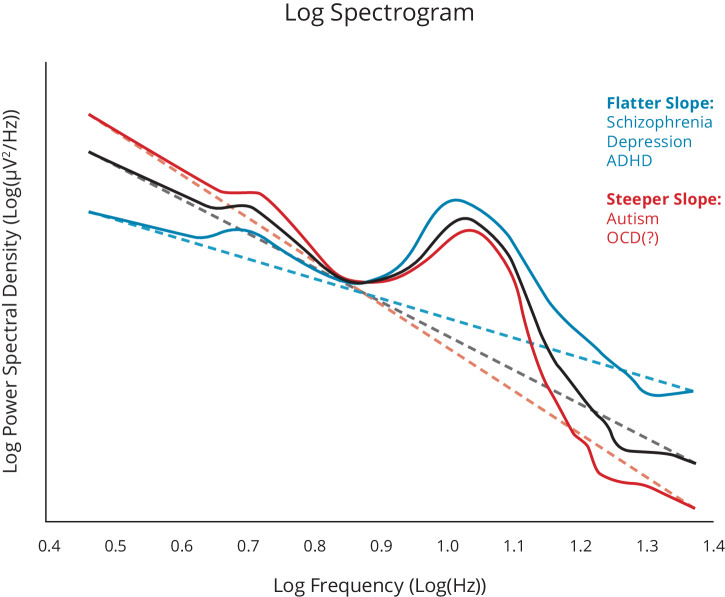
Schematical illustration of possible modulations of 1/f noise in a log-log plot of the power spectrum (fictive data). Psychiatric conditions with possibly show lower or higher “noise” are shown in different colours.

Considering the different approaches to better conceptualize metacontrol on a neural level, current findings regarding 1/f slopes seem to largely match the characterization in our examples above (see Fig. 5). At the persistence end, steeper slopes in preterm infants have been found to predict autism risk at 3 years of age^85,86^.

However, a recent study found no slope differences in the resting state (RS) spectrogram of individuals with OCD versus healthy controls^87^. Conversely, shallower RS slopes have been observed in individuals with schizophrenia^88,89^, but see^90^. In depression, shallower slopes have been found during sleep^91^, and treatment response to brain stimulation seems to be associated with a slope increase in this population^92,93^. A flattened resting state slope has also been found in ADHD, which seems to develop from an initially steeper slope in this population through an exaggerated age-related flattening^94,95^—again highlighting the importance of a sufficiently dynamic and developmental perspective. In children with ADHD, a flatter slope has been observed during response inhibition, which is normalized using treatment with methylphenidate^96^. In keeping with a view on metacontrol as the ability to *shift* persistence/flexibility in light of circumstantial demands, cognitive load, and stimulus salience had less of a modulatory effect on an overall shallower slope in children with ADHD in a visual oddball task^97^. Of note, this research is in its infancy. The 1/f slope is dependent on task context, developmental stage, and electrode location. It may have differential functional significance across different portions of the frequency spectrum—all factors that are yet to be systematically investigated. Intriguingly, considerable evidence suggests that not the broad-band activity but low-frequency oscillations, particularly in the theta band, are likely important when understanding psychopathologies^16,98^ and are also of considerable importance for cognitive control processes^31,99^ that can be described along a persistence-flexibility continuum^7^.

There is very little literature on the use of HMMs to characterize the metacontrol-defined persistence/flexibility axis in neuropsychiatric disorders and even less on aspects, such as chaotic itinerancy and phase precession. Shappell et al. ^100^ analysed fMRI data using Hidden semi-Markov models and demonstrated that children with ADHD spend more time in one well-connected brain state with the highest global and local efficiency and lowest modularity, and less time in default mode network, and task-relevant networks as compared to typically developing children. Similarly, Kottaram et al. ^101^ demonstrated that the DMN was less frequently visited in people with schizophrenia with shorter durations per visit, and Lin et al. ^102^ observed shorter lifetimes across many different brain states in autism spectrum disorder. Importantly, these three studies employed functional MR imaging data and are thus intrinsically limited in their temporal resolution. In this framework, neural mass models describe the dynamics of populations of neurons and how they generate observable signals. The possibility to generate simulated data and test different hypotheses can help researchers gain insights into the brain information process and understand how changes in neural dynamics relate to cognitive functions and disorders. In particular next-generation neural mass models^70,71,103–106^ allow for the generation of frequencies in different ranges, as well as phase-amplitude coupling or cross-frequency coupling. In this direction, choosing a proper set of parameters that allow for the emergence of low-frequencies in the theta/delta range will help to verify the hypothesis of a smaller presence of theta/delta rhythms in OCD/ADHD. On the other hand, the suggested mean-field description relates structural and functional connectivity, as mentioned in the previous Section, showing how changes in the structural connectivity are reflected in the macroscopic dynamics^107^. Thus, we may hope to employ it in discerning whether or not the dichotomy stability/flexibility in OCD/ADHD is explained by changes in structural connectivity.

## Conclusion

The human brain demonstrates remarkable flexibility and persistence in adapting to various situations, known as executive functions, crucial for goal-directed behavior and mental health. Recent studies suggest that a concept called “metacontrol” overlays these functions, enabling individuals to adjust between persistent and flexible cognitive styles depending on the situation. In this paper, we have identified multiple avenues to conceptualize metacontrol on a neural level. We highlighted the potential of aperiodic broadband EEG activity as a means to capture persistence. Yet, as a characteristic of the frequency spectrum amplitude, we lose dynamic and phase-based information. Therefore, we highlighted the potential of chaotic itinerancy, phase precession and of hidden Markov models, and phase-based measures to capture persistence or flexibility at a very high temporal resolution. Finally, whereas signal processing techniques may lead to novel biomarkers, neurocomputational models provide a theoretical framework for understanding the dynamics of neural populations and may bring insights into why specific neurophysiological features are altered. We highlighted recent developments in these models that now enable us to simulate the 1/f power spectrum and offer insights into the dynamics of functional connectivity networks and phase stability and their role in health and disease.

## Data Availability

No data were collected for this article.
